# Nitrogen dioxide levels estimated from land use regression models several years apart and association with mortality in a large cohort study

**DOI:** 10.1186/1476-069X-11-48

**Published:** 2012-07-18

**Authors:** Giulia Cesaroni, Daniela Porta, Chiara Badaloni, Massimo Stafoggia, Marloes Eeftens, Kees Meliefste, Francesco Forastiere

**Affiliations:** 1 Department of Epidemiology, Lazio Regional Health Service, Via S. Costanza 53, 00198, Rome, Italy; 2 Institute for Risk Assessment Science, Utrecht University, Utrecht, The Netherland

**Keywords:** Land use regression, Air pollution, Mortality, Long-term exposure, Nitrogen dioxide

## Abstract

**Background:**

Land Use Regression models (LUR) are useful to estimate the spatial variability of air pollution in urban areas. Few studies have evaluated the stability of spatial contrasts in outdoor nitrogen dioxide (NO_2_) concentration over several years. We aimed to compare measured and estimated NO_2_ levels 12 years apart, the stability of the exposure estimates for members of a large cohort study, and the association of the exposure estimates with natural mortality within the cohort.

**Methods:**

We measured NO_2_ at 67 locations in Rome in 1995/96 and 78 sites in 2007, over three one-week-long periods. To develop LUR models, several land-use and traffic variables were used. NO_2_ concentration at each residential address was estimated for a cohort of 684,000 adults. We used Cox regression to analyze the association between the two estimated exposures and mortality.

**Results:**

The mean NO_2_ measured concentrations were 45.4 μg/m^3^ (SD 6.9) in 1995/96 and 44.6 μg/m^3^ (SD 11.0) in 2007, respectively. The correlation of the two measurements was 0.79. The LUR models resulted in adjusted R^2^ of 0.737 and 0.704, respectively. The correlation of the predicted exposure values for cohort members was 0.96. The association of each 10 μg/m^3^ increase in NO_2_ with mortality was 6 % for 1995/96 and 4 % for 2007 LUR models. The increased risk per an inter-quartile range change was identical (4 %, 95 % CI:3–6 %) for both estimates of NO_2_.

**Conclusions:**

Measured and predicted NO_2_ values from LUR models, from samples collected 12 years apart, had good agreement, and the exposure estimates were similarly associated with mortality in a large cohort study.

## Background

Several cohort studies from Europe and North America have suggested an association between long-term exposure to air pollution and adult mortality for natural causes, cardiovascular diseases, respiratory diseases, and lung cancer [1-14]. Despite the number of studies produced so far, however, the evidence that traffic-related air pollution is related to mortality has been considered suggestive but not yet sufficient [15].

A specific challenge in studying the effect of long term exposure to ambient air pollution is the exposure assessment. The possible approaches to consider intra-urban air pollution contrasts include the use of dispersion models, interpolation methods, Geographic Information System (GIS) proxy measures of traffic exposure, and land use regression models. The latter methodology was developed in the SAVIAH (Small Area Variation In Air quality and Health) study and has become a commonly used approach [16-20]. The method consists in predicting pollution concentrations at a given site using surrounding characteristics: geographical and land-use variables such as altitude, population density, meteorology, GIS and traffic flows variables.

The application of the results of the land use regression model faces a relevant methodological problem related to the timing of the measurements of exposure. Since the methodology is relatively recent and because monitoring is costly, the same estimates from land use regression models are often used to estimate exposure contrasts in different time periods. The basic assumption is that spatial patterns of air pollution change slowly in a city and that exposure assessment performed today can be a good surrogate of exposure occurring in the past or in the future. However, few studies has challenged this assumption, by comparing the performance of land use regression models over long time periods with measured and predicted levels of pollutants within the same area [18,19]. In addition, there are no available studies that have evaluated the performance of the exposure estimates from land use regression models taken several years apart for a resident population, or have investigated the association with mortality.

The present study was designed to fill these gaps. In particular, we developed two land use regression models for NO_2_ in the city of Rome with a time span of 12 years, and compared them in terms of observed and predicted values, exposure predictions, and association with mortality for natural causes in a large cohort of adult residents followed from 2001 to 2006.

## Methods

### The setting

Rome is the largest Italian city with a population of 2.8 million inhabitants on a surface of 1,290 km^2^. It is a radial city, and the urban development in Rome took place gradually from the centre to the suburbs, with significant urbanization in the 1930s, after the second world war, and in the 1990s [21]. The city is divided into 5,500 census blocks with an average population of 470 inhabitants.

### Air pollution measurements in 1995–96

Nitrogen dioxide (NO_2_) was measured at 70 schools in a cross sectional study on children conducted in 1995 (Italian Study on Respiratory Diseases and Environment), the Italian part of the International ISAAC study [22]. The objective of the measurement campaign was to estimate average NO_2_ exposure at schools in Rome and the 70 schools were a random sample of all primary and junior high schools of the city (stratified by the 20 city districts). Three Palmes diffusion tubes, manufactured by the Emilia–Romagna Environmental Protection Agency, measured outdoor air pollution in each location simultaneously over three one-week periods in June 1995, November 1995, and March 1996 [23]. The tubes were placed outside the school, two meters above ground, and the mean of the measurements for the three tubes was considered. The school mean NO_2_ concentration over the three periods was considered as an estimate of the annual mean level. Only sites that had complete data coverage over the three periods were included, thus 67 sites remained for the analysis [23]. The locations were 44 urban background sites, and 23 traffic sites.

### Air pollution measurements in 2007

In 2007, we measured NO_2_ concentrations using Ogawa passive samplers at 78 sites during three one-week periods in February, May, and October. As in the previous survey, the samplers were positioned within two days and collected exactly after one week. We selected the same locations used in 1995/96, and we added 11 sites to increase the variability of the measurements (3 urban background, 7 traffic sites, and 1 regional background site). In fact, we realized that schools selected in 1995/96 were not enough to represent the high variability of NO_2_ in Rome, and a previous LUR developed with 1995/96 measurements tended to underestimate high values of NO_2_[23]. In 27 locations, we used the same Palmes tubes and Ogawa samplers to compare the different measures and calculate a correction factor between the two methodologies. An Ogawa badge is a passive sampler that collects nitrogen oxides through diffusion on a pre-coated filter [19,24]. The filter is removed in the laboratory and analyzed with a spectrophotometer for nitrites using a Saltzman reaction. Five lab blanks from each measurement campaign were kept at our department. All the analyses of the Ogawa samplers were performed at the Institute for Risk Assessment Science of Utrecht University [19]. Field blanks and field duplicates were taken to document detection limits and precision of each measurement campaign. The Palmes tubes were analyzed by the Emilia-Romagna Environmental Protection Agency.

**Figure 1:**
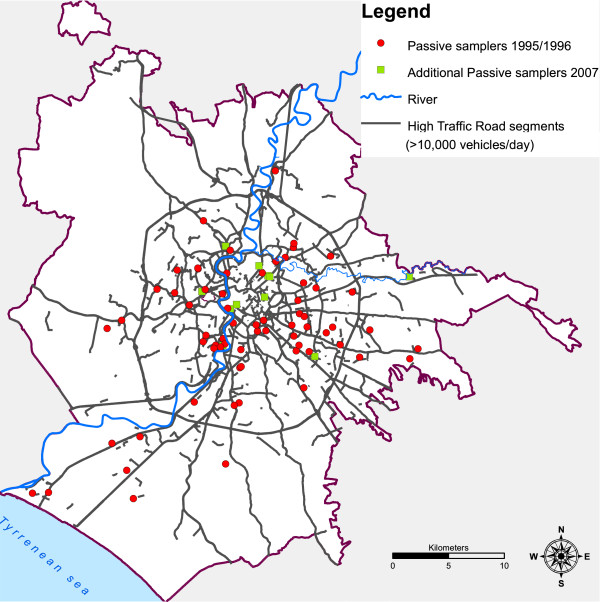
**Location of NO**_**2**_**measurements, and high traffic roads (>10,000 vehicles/day), Rome.**

We assigned to each sampling location a single NO_2_ level, the mean of the three measurements. The map of Rome with the sites of measurement is presented in Figure 1.

### Land use and traffic variables

Several land-use and traffic variables were available to characterize the locations used for air pollution measurements. Traffic flows of 2005 (only for roads with >4,000 vehicles per day) were provided by the Municipality of Rome and were used for both models. For each location, we used the variables already chosen to develop a previous LUR model for Rome [23]: the proximity to green urban areas (a binary variable, which indicated if the census block was adjacent to a green urban area, selected using CORINE land cover data), the altitude, the size of the census block, the number of residents in the census block, and the inverse of the population density for the census block (m^2^ per person). To increase our predictive ability, we added as linear terms the distance from the city centre and the geographical coordinates (X and Y). The city center has the highest traffic and population density levels and it is expected that air pollution tends to be higher in the center and lower in the suburbs so that the distance variable should capture this aspect. On the other hand, the orography of the area is peculiar (the Mediterranean Sea at South-West and the hills towards East and North-East ) and the prevalent wind direction tend to have a West- East direction. Therefore, the X and Y coordinates may capture the influence of orography and wind. In addition, we considered three GIS variables to characterize the traffic that surrounds each location: meters of high traffic road (more than 10,000 vehicles/day) within a buffer of 150 meters, traffic density (number of cars multiplied by the length of roads within 150 metres divided by the buffer area), distance from the closest high traffic road. For sake of simplicity, only one buffer was chosen (150 metres) considering the decay gradients of NO_2_ with the distance to high traffic roads [15].

We collected and stored all geographical variables using ArcGis 9.1 (ESRI, Redlands, California, USA). We used the Word Geodetic System of 1984 with the Universal Transverse Mercator 33 N (WGS84 UTM33N) as the coordinate system and map projection.

### The Rome Longitudinal Study (RoLS)

The Rome Longitudinal Study is a cohort based on the Rome Municipal Register’s data, described in detail elsewhere [25]. Briefly, it is a closed prospective cohort of residents of Rome on the 21^st^ October 2001 with a mortality follow up to the end of 2006. All 2001 residential addresses were geocoded using the Italian road network (Tele Atlas, Italy). Several individual socio-demographic variables at baseline were available for each subject of the cohort (including the residential history, education, occupation, place of birth, marital status). In addition, we used a small-area (census block, average population: 470 inhabitants) composite index of socioeconomic position (SEP), built with 2001 Census data [26], to better characterize social deprivation.

We selected all adults aged 45–80 years in 2001 who had not changed their residence in the five years preceding enrolment and during the follow-up. We linked subjects to the Regional Mortality Registry, and collected the date of death and the underlying cause of death (coded to the International Classification of Diseases (ICD) revision 9). We evaluated their vital status until December 2006 and analysed the association between NO_2_ exposure (estimated with the two LUR models) and natural mortality (ICD-9 < 800), taking into account individual characteristics (age, gender, marital status, place of birth, education, occupation) and area-based socioeconomic position, as a surrogate measure of lifestyles and access to health services.

### Statistical analysis

We used data from the 27 sites with both Palmes tubes and Ogawa samplers in 2007, to compare the measures of ambient NO_2_ performed with different methods. Therefore, we applied a correction factor derived from a linear regression to all 1995/96 Palmes tube measurements in order to align them with the measurements collected with the Ogawa passive samplers.

We evaluated the correlation between NO_2_ measurements in 1995/96 and 2007 with the Pearson correlation coefficient, and their agreement using the Bland-Altman plot. To build the period-specific LUR models, the association between the logarithmic transformation of NO_2_ concentrations and each land-use or traffic variable was assessed by univariate and multiple linear regressions. We used the logarithmic transformation because the log-normal NO_2_ distribution. The final model was constructed using a manual backward stepwise elimination procedure (p > 0.20): we eliminated from the model the predictors one-by-one on the basis of the highest p-value. When one of the coordinates was selected, we kept into the model the other coordinate independently on its p-value. Variables with a regression slope of opposite sign to what was expected were not included; in other words, we excluded all the variables known to be positively associated to air pollution, which seemed to have a negative effect instead.

The validity of the regression models was evaluated by leave-one-out cross-validation, i.e. by systematically subtracting each of the data points from the model one by one, and then comparing the predicted value for each specific point with the measured level at the location without that value in the model [27]. We plotted measured and predicted levels from the cross-validation in 1995/96 and 2007, and we calculated the root mean square errors (RMSE) and R^2^ of regression analysis between measured and estimated concentrations. We checked each model for influential observations, performing the Cook’s distance, for multicollinearity by estimating the variance inflation factor (VIF), and we calculated the Moran’s Index of residuals, to check their spatial autocorrelation.

We assessed the ability of LUR models to predict concentrations both prospectively (using the 1995/96 model to predict measured concentrations in 2007), and retrospectively (using the 2007 model to predict 1995/96 measured concentrations).

We estimated the NO_2_ concentrations for all residence addresses of the participants of the Rome Longitudinal Study using the two LUR models. We used the Pearson correlation coefficient to measure the correlation between the results of the LUR models from 1995/96 with those from 2007 at all the residential addresses of subjects enrolled in the cohort. We used a test for trend across ordered groups for comparing levels of NO_2_ among age and socioeconomic categories, and a t-test for comparing the exposure levels between genders.

Finally, we applied Cox proportional hazard regression models to analyse the association between NO_2_ exposure (from both LUR models) and natural mortality, while adjusting for age, gender, marital status, place of birth, education, occupation and area-based socioeconomic position. We categorized the predicted NO_2_ levels into quintiles and into four pre-specified categories (<=35 μg/m^3^, 35–45 μg/m^3^, 45–50 μg/m^3^, >50 μg/m^3^). We calculated hazard ratios of natural mortality for 10 μg/m^3^ NO_2_ and for interquartile range.

## Results

The Pearson correlation coefficient between the measurements taken in 2007 (at the same sites) with Palmes tubes and those taken with Ogawa samplers in 2007 was 0.92. The following equation, from a linear regression (R^2^ = 0.84), was used to calibrate the measures taken in 1995/96 with those taken in 2007: NO_2-Ogawa badge_ = 0.68*NO_2-Palmes tube_ + 13.53

Mean NO_2_ concentration was 45.4 μg/m^3^ (SD 6.9) in 1995/96. The mean values were rather similar in 2007, 44.6 μg/m^3^, but the standard deviation increased (SD 11.0) partly due to the additional 11 sites added to the analysis. Restricting the calculation to the original 1995/96 sites, the mean value for 2007 was 42.7 μg/m^3^ (SD 9.1). The NO_2_ concentrations slightly decreased both in traffic and urban background sites (Table 1). The description of land-use and traffic variables and mean and standard deviation (SD) of NO_2_ measures in 1995/96 and 2007 is presented in Additional file 1: Table S1.

**Table 1:** **Nitrogen dioxide (NO**_**2**_**) concentrations by measurement campaign and site type (μg/m**^**3**^**)**

		**Urban Background sites**	**Traffic sites**	**Total**
1995/96 measurements	Number of sites	44	23	67
	Mean (sd)	43.7 (6.0)	48.6 (6.3)	45.4 (6.5)
	Range	30.0-57.3	35.4-63.5	30.0-63.5
2007 measurements in the same locations	Mean (sd)	40.5 (7.5)	46.8 (10.5)	42.7 (9.1)
	Range	25.0-55.6	29.0-70.2	25.0-70.2
2007 measurements	Number of sites	47	30	78
	Mean (sd)	40.8 (7.6)	51.0 (12.5)	44.6 (11.0)
	Range	25.0-55.6	29.0-72.6	25.0-72.6

### Land use regression models

The univariate and multivariate association between land-use and traffic variables and the log of NO_2_ concentration at the 67 sites in 1995/96 and at the 78 sites in 2007 is shown in Table 2. Multiple regression models indicated that the following variables predicted measured NO_2_ in 1995/96: altitude, geographical coordinates, distance from the city centre, inverse population density of the census block, traffic density in the 150 m buffer around the site, and distance from the closest road with more than 10,000 vehicles daily. The best-fitting multiple regression model resulted in a determination coefficient (R^2^) of 0.737, and a root mean square error (RMSE) of 0.076 (i.e. 1.09 μg/m^3^).

**Table 2:** **Association between logarithm of nitrogen dioxide (NO**_**2**_**) concentrations and land-use variables, from univariate analysis and multiple linear regression model. Rome 1995/96 and 2007**

			**1995/96**			**2007**		
Land-use variables	Slope^a^	p value	Slope^b^	p value	Slope^a^	p value	Slope^b^	p value
*Intercept*			*(24.5)*	*0.023*			*(21.8)*	*0.199*
Proximity to a green urban area								
No	-							
Yes	−0.0067371	0.895			−0.0482182	0.517		
Altitude (m)	−0.0009000	0.239	−0.0013084	0.002	−0.0017940	0.138	−0.0025286	0.001
GIS Coordinate x (m)	0.0000031	0.233	0.0000031	0.054	0.0000060	0.148	0.0000069	0.009
GIS Coordinate y (m)	0.0000090	0.002	−0.0000046	0.049	0.0000164	0.001	−0.0000042	0.252
Distance from the city centre (m)	−0.0000218	<0.001	−0.0000176	<0.001	−0.0000355	<0.001	−0.0000301	<0.001
Number of residents in the census block	−0.0000500	0.295			−0.0001610	0.004		
Size of the census block (m^2^)	−0.0000004	<0.001			−0.0000004	<0.001	−0.0000002	0.001
Inverse population density (m2/person)	−0.0002000	<0.001	−0.0001024	0.004	−0.0000072	0.248		
Meters of high traffic road in a 150 m buffer	0.0003840	0.001			0.0005537	<0.001	0.0003620	<0.001
Traffic density in a 150 m buffer (cars/m)	0.0008000	0.001	0.0003290	0.032	0.0013315	<0.001		
Distance from the closest high traffic road (m)	−0.0005000	<0.001	−0.0002021	0.003	−0.0006183	<0.001		
*Adjusted R-square*			*0.737*				*0.704*	

^a^The estimate derived from a simple linear regression model with only one independent variable and logarithm of NO_2_ concentration (μg/m^3^) as dependent variable.

^b^The estimate derived from a multiple linear regression model with logarithm of NO_2_ concentration (μg/m^3^) as dependent variable.

The following variables were associated with NO_2_ concentrations in 2007: altitude, the X-coordinate, distance from the city centre, size of the census block, and meters of high traffic road within 150 m of the site. The best-fitting multiple regression model resulted in a determination coefficient (R^2^) of 0.704, and a RMSE of 0.132 (i.e. 1.14 μg/m^3^).

For both models there was no evidence of multicollinearity among the chosen variables or of influential observations: the mean variance inflation factor was 1.55 (the maximum was 2.08 for Y-coordinate) in 1995/96 and 1.39 (the maximum was 1.92 for Y-coordinate) in 2007, the Cook’s distance was less than 0.3 in both cases. There was no evidence of spatial autocorrelation of residuals: the p value of Moran’s Index was 0.300 in 1995/96 and 0.218 in 2007.

### Cross-validation of the two models

For the first period, the determination coefficient (R^2^), the adjusted-R^2^ and the root mean square error of regression analysis between measured and estimated concentrations were 0.67, 0.66, and 3.24 respectively.

In 2007, the determination coefficient (R^2^), the adjusted-R^2^ and the root mean square error of regression analysis between measured and estimated concentrations were 0.61, 0.61, and 5.38 respectively.

### Comparison of the 1995/96 and 2007 observed and predicted levels

**Figure 2:**
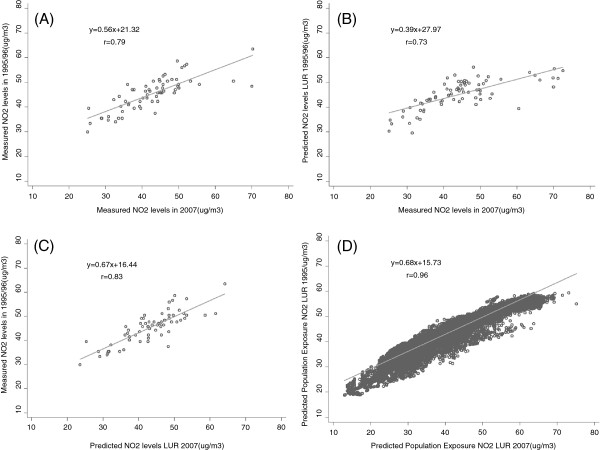
**Comparison of the measurements of NO**_**2**_**concentrations taken at the same locations in the two study periods. (A)**, the ability of 1995/96 model to predict 2007 measurements **(B)**, the ability of 2007 LUR model to predict 1995/96 measurements **(C)**, and the comparison of predicted values of the two LUR models at the addresses of the study population **(D).**

Figure 2 shows the scatter plots of the comparison of the measurements of NO_2_ concentrations taken at the same locations in the two study periods (A), the ability of 1995/96 model to predict 2007 measurements (B), the ability of 2007 LUR model to predict 1995/96 measurements (C), and the comparison of predicted values of the two LUR models at the addresses of the study population (D). The Pearson correlation coefficient between the two measurements of ambient NO_2_ was 0.79, it was higher for urban background sites (0.83) than for traffic sites (0.68).

The predictions of 1995/96 LUR model and measurements in 2007 had a correlation of 0.73, while the 2007 LUR predicted levels had a correlation with 1995/96 measurements of 0.83. When we applied the estimates of NO_2_ concentration derived from the two LUR models to the residential addresses of the 684,204 subjects enrolled into the study, the correlation between the estimated measures in the two periods was 0.96.

### NO_2_ exposure for cohort members

**Figure 3:**
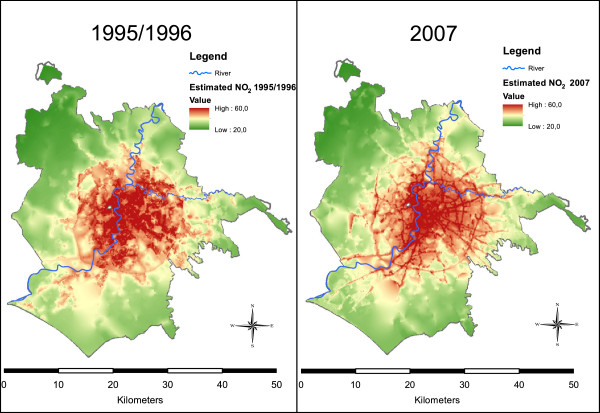
**Maps of Rome with predicted NO**_**2**_**levels in 1995/96 and in 2007.**

Figure 3 shows the maps of levels of NO_2_ from the two land use regression models in Rome. The socio-demographic characteristics of the study population and the levels of exposure according to the 1995/96 and 2007 models are presented in Table 3. According to the 1995/96 model, the 684,204 cohort members were exposed to a mean level of 45.7 μg/m^3^ (SD 5.9), the level was slightly higher in women than in men (p < 0.001), and was higher in the elderly than in the 45–64 year old population (p-trend < 0.001), and in subjects living in high compared to low socioeconomic position census blocks (47.2 vs. 42.9 μg/m^3^, p-trend < 0.001). According to the model developed with 2007 data, similarly to the 1995/96 model, the mean exposure was 43.9 μg/m^3^, and levels of exposure increased with increasing age class (p-trend < 0.001) and increasing socioeconomic position (p-trend < 0.001).

**Table 3:** **Nitrogen dioxide (NO**_**2**_**) exposure levels predicted in 1995/96 and 2007 according to population characteristics**

		**NO**_**2**_						
**Variable**	**N**	**mean**	**sd**	**Q25**	**Q50**	**Q75**	**min**	**max**
**1995/96**								
All	684,204	45.7	5.9	42.6	46.6	49.8	18.8	59.3
Gender								
Men	306,018	45.4	6.0	42.2	46.4	49.7	18.8	59.3
Women	378,186	45.8	5.9	42.8	46.8	49.9	18.8	59.3
Age (years)								
45-64	418,241	45.3	6.1	42.0	46.2	49.6	18.8	59.3
65-74	186,614	46.0	5.8	43.2	47.0	50.0	18.8	59.3
75-80	79,349	46.8	5.4	44.2	47.7	50.4	18.8	59.1
Socioeconomic position (SEP)								
High	138,457	47.2	4.2	44.5	47.6	50.1	24.3	58.0
medium-high	142,446	47.1	5.2	44.5	47.9	50.8	22.0	59.1
Medium	139,072	46.5	5.7	43.3	47.4	50.4	20.1	59.3
medium-low	137,017	44.3	6.7	39.9	45.6	49.1	18.8	58.8
Low	127,212	42.9	6.4	39.3	43.8	47.6	18.8	58.8
**2007**								
All	684,013	43.9	8.3	39.0	44.8	49.4	13.0	75.2
Gender								
Men	306,018	43.6	8.4	38.6	44.5	49.1	13.0	73.1
Women	378,186	44.1	8.3	39.4	45.0	49.6	13.0	75.2
Age (years)								
45-64	418,241	43.4	8.4	38.2	44.2	49.0	13.0	75.2
65-74	186,614	44.4	8.2	39.9	45.3	49.7	13.0	73.1
75-80	79,349	45.5	7.9	41.7	46.1	50.5	13.1	69.4
Socioeconomic position (SEP)								
High	138,457	46.0	6.3	42.2	46.0	49.6	19.8	68.6
medium-high	142,446	45.8	7.8	41.9	46.2	50.9	18.2	75.2
Medium	139,072	44.9	8.5	40.1	45.7	50.8	14.6	73.1
medium-low	137,017	42.1	9.2	34.9	43.0	48.0	13.0	67.5
Low	127,212	40.4	8.2	35.3	41.2	46.3	13.0	65.1

### Association with natural mortality

During the study period, a total of 45,006 deaths from natural causes occurred in the cohort. Table 4 shows the results of the Cox regression analyses adjusted for several confounders. When we considered the quintiles of the two distributions as the relevant exposures, we obtained very similar results of increased mortality from natural causes with increasing quintiles of NO_2_ at residence (HR = 1.10, 95%CI:1.06-1.13 in the 5^th^ quintile compared to the 1^st^ for 1995/96 estimates, and HR = 1.11, 95%CI:1.07-1.14 in the 5^th^ quintile compared to the 1^st^ for 2007 estimates). There was a similar association with natural mortality even when we used a 4-category variables with a 12 % increased risk of dying (95%CI: 8–18 %) for those with a concentration of NO_2_ at residence of more than 50 μg/m^3^, compared to those exposed to less than 35 μg/m^3^. The increased mortality risk for an increase of 10 μg/m^3^ of NO_2_ concentration in 1995/96 was 6 % (95%CI:4–8 %), while using NO_2_ estimates for 2001 it was 4 % (95%CI:3–5 %). This result was related to the wider variability of NO_2_ concentrations in 2007; in fact the hazard ratios of mortality for an increase of NO_2_ corresponding to the inter-quartile range was 1.04 (95%CI: 1.03-1.06) for both periods.

**Table 4:** **Adjusted hazard ratios (HR) of the association between nitrogen dioxide (NO**_**2**_**) estimated from the 1995/96 and the 2007 LUR models and natural mortality. Rome 2001-2006**

	**1995/96 LUR model**					**2007 LUR model**				
	**Population**	**HR**	**95%CI**			**Population**	**HR**	**95%CI**		
Quintiles of NO_2_										
1	136,842	1.00				136,841	1.00			
2	136,840	1.04	1.01	-	1.07	136,842	1.06	1.03	-	1.09
3	136,885	1.08	1.05	-	1.11	136,846	1.08	1.04	-	1.11
4	136,829	1.08	1.04	-	1.11	136,843	1.09	1.06	-	1.13
5	136,808	1.10	1.06	-	1.13	136,832	1.11	1.07	-	1.14
p-trend^§^		<0.001					<0.001			
Categories of NO_2_										
<=35 μg/m^3^	44,357	1.00				103,836	1.00			
35-45 μg/m^3^	217,880	1.05	1.01	-	1.10	246,148	1.07	1.04	-	1.10
45-50 μg/m^3^	259,318	1.10	1.05	-	1.14	181,160	1.10	1.07	-	1.14
>50 μg/m^3^	162,649	1.13	1.08	-	1.18	153,060	1.12	1.08	-	1.16
p-trend^§^		<0.001					<0.001			
Effect per linear increase in NO2										
per 10 μg/m^3^		1.06	1.04	-	1.08		1.04	1.03	-	1.05
per IQ range*		1.04	1.03	-	1.06		1.04	1.03	-	1.06

HR, Hazard Ratios, adjusted for age, sex, marital status, place of birth, education, occupation, and area-based socioeconomic position.

* The IQ ranges are 7.28 μg/m^3^ and 10.37 μg/m^3^, in 1995/96 and in 2007, respectively.

§ Wald test.

## Discussion

We documented the stability of NO_2_ measurements and predicted levels of exposure by two LUR models twelve years apart. The NO_2_ concentrations measured in the same locations had a good agreement, showing the stability of spatial contrast. This study shows that 74 % and 70 % of the NO_2_ variability in Rome in 1995/96 and in 2007, respectively, is explained by land use and traffic variables. The 2007 LUR model was able to predict NO_2_ levels measured 12 years before in a better way than what the 1995/96 model was able to do prospectively (r = 0.83 vs. 0.73). When we applied the estimated NO_2_ from the two LUR models to residential addresses of the large cohort members, the correlation was extremely high (0.96). The association between NO_2_ exposure at residence and mortality from 2001 to 2006 was very similar using the 1995/96 and the 2007 models.

The choice of sites for measurements was driven by the 1995/96 campaign which aimed to investigate the level of exposure of children at school. The schools, randomly selected, were placed mostly in urban background sites (44/67). We did notice that the prediction from the LUR model underestimated to some extent the highest levels of NO_2_[23]. On the other hand, 17 % of the residents in the city live at less than 50 metres from a high traffic road, which means that a significant proportion of residents is not represented by urban background locations [25]. Therefore, to better characterize the variability of NO_2_ in the city we added 11 sites (7 high traffic locations) in the second measurement campaign. Potential consequences of this choice were a lower R^2^ of the 2007 LUR model and a poorer ability of the 1995/96 model to predict the 2007 measurements compared to the performance of the 2007 model to predict the observations retrospectively. On the other hand, the R^2^ of the models were comparable to those developed in other settings that went from 0.51 to 0.90 [17], and were better than the one we developed a few years ago (R^2^ was 0.69) when data on vehicular traffic were not available [23].

A slight decrease in air pollution was noticed when comparing the results of the two surveys, and it occurred both in traffic and in urban background sites. Data from fixed monitors of the Regional Environmental Protection Agency show in the period 1999–2008 a small decrease in NO_2_ concentrations in traffic sites, and a stability in urban background sites [28].

Few studies have been conducted on the stability of small-area spatial contrasts over time using LUR models and they corroborate our findings. In Oslo, Madsen and colleagues found a good agreement in spatial contrasts over a three year period. [18] In the Netherlands, Eeftens and colleagues showed good agreement in measured and modelled levels of NO_2_ eight years apart [19].

The prediction of NO_2_ levels to the studied population indicates that traffic-related air pollution in Rome is not evenly distributed throughout the population: we found higher levels of exposure in the oldest age group, and in the higher socioeconomic compared to the lower socioeconomic census blocks. This is not surprising as other local studies have shown the same pattern, which is related to the urbanization history of Rome, with the elderly and well-off population more likely to live in the prestigious and well-travelled city centre than in the periphery [25,29]. Although the common belief is that low socioeconomic groups of the population are disadvantaged from all points of view, air quality included, this idea has been contradicted also by studies conducted in Canada and the Netherlands [6,30].

The mortality - NO_2_ association was identical using the two LUR models in all circumstances: when using quintiles of the distribution, categories of NO_2_ concentration, and the inter-quartile range. However, the effect was slightly lower with the 2007 model compared to 1995/96 when we calculated the association with natural mortality for a 10 μg/m^3^ increase in NO_2_ and this is attributable to the lower estimated variability in 1995/96. Nevertheless, it is worth noting that our estimates of the strength of the association between NO_2_ and mortality were similar to findings in other settings [1,3,5].

This study has some limitations. We used two different methods to measure NO_2_ concentrations: Palmes tubes and Ogawa badges. Although we took it into account, calibrating the 1995/96 levels, we introduced some level of error: 16 % of variance in measurements taken using Ogawa samplers was unexplained by Palmes tube measurements. Moreover, we assigned a correction factor based only on one week of measurements, while it could change in different weather conditions. In each campaign we used as a single measure of NO_2_ concentration the mean of the three measurements in the same site, without taking account of variation during the year. The mean concentration of NO_2_ during the first measurement period in urban background fixed monitors was 47.0 μg/m^3^, comparable to our mean of 45.5 μg/m^3^[23]. Data were not available to adjust 1995/96 measurements for temporal trends and for this reason we have not done it neither for 2007. However, during the three weeks of measurements in 2007 at the city background monitoring station the average concentration of NO_2_ was 44.9 μg/m^3^ (SD 14.6) and was comparable to the annual average concentration we estimated (43.9 μg/m^3^, SD 14.6). An important limit of our models is that traffic data were available only for major roads (i.e. >4000 vehicles/day), and they were estimated using an integrated data monitoring and evaluation system developed under the Heaven European project (heaven.rec.org). Moreover, period-specific data on traffic were not available as we used for both models the same 2005 traffic data. The only time-specific variables were those related to population. For the cohort analysis, we did study subjects who did not move since five years before the enrolment to the end of the follow-up. This is a strength of the study and in Rome the majority of adult population is rather stable and the percentage of those aged 45–80 years who change the address during ten years is 15 % and decrease with increasing age. For these reasons we believe that our mortality analysis is valid. The cohort we used is based on administrative data, and information on important risk factors and potential confounders such as obesity, smoking, diet are not available. However, we were interested on comparing the effect of the two estimates from LUR models on the same population, than measuring the effect estimates of air pollution exposure on mortality. In fact we wanted to investigate to what extent we can use exposure models based on measurements taken after the settlement of the epidemiologic study.

## Conclusions

Rome is a city with slow changes in terms of urbanization and traffic volumes, hence it is possible to use current LUR models to estimate contrasts in air pollution exposure even if they refer to a long time before. We have shown that land use regression models for NO_2_, developed with independent measurement data collected 12 years apart, produced very similar results and were similarly associated with increased mortality in a large population study.

## Abbreviations

LUR, Land use regression; NO2, Nitrogen dioxide; SEP, Socioeconomic position; VIF, Variance inflation factor.

## Competing interests

None of the authors have any competing interests to declare.

## Authors’ contributions

DP, KM, ME conducted and analysed the 2007 measurements. DP, GC and FF designed the study. CB conducted GIS analysis. GC performed the statistical analyses. GC and FF wrote the manuscript. All authors provided edits and comments to the manuscript. All authors read and approved the final manuscript.

## Supplementary Material

**Additional file 1:** **Table S1.** Description of land-use and traffic variables and mean and standard deviation (SD) of NO_2_ measures in 1995/96 and 2007. Click here for file
